# Bilateral acute intermediate uveitis following intrauterine Chitosan tamponade: a case series

**DOI:** 10.1186/s12348-025-00548-9

**Published:** 2025-12-24

**Authors:** David Beckers, Uwe Pleyer, Lena Beckers, Holger Maul, Mascha Lüder, Ulrich Schaudig, Birthe Stemplewitz

**Affiliations:** 1 Precise Vision Ophthalmologists, Rheine, Germany; 2 Department of Ophthalmology, Charité — Universitätsmedizin Berlin, Corporate Member of Freie Universität Berlin and Humboldt-Universität zu Berlin, Berlin, Germany; 3 Department of Obstetrics and Gynecology, Asklepios Klinik Barmbek, Hamburg, Germany; 4 Department of Ophthalmology, Asklepios Klinik Barmbek, Hamburg, Germany

**Keywords:** Case series, Chitosan, Inflammation, Intrauterine tamponade, Obstetrics, Intermediate uveitis, Postpartum hemorrhage

## Abstract

**Background:**

Postpartum hemorrhage is a leading cause of maternal morbidity and mortality, with intrauterine tamponade techniques increasingly employed in its management. Chitosan-based products, such as Celox^®^, have been introduced for their hemostatic properties. However, their potential systemic effects remain insufficiently understood. This case series describes six patients who developed acute bilateral intermediate uveitis shortly after receiving intrauterine Chitosan tamponade during obstetric care.

**Main body:**

A retrospective review was conducted on six postpartum patients who developed acute bilateral intraocular inflammation following the use of intrauterine Chitosan tamponade. Clinical data, laboratory findings, and ophthalmological assessments were analyzed. All six women presented with bilateral anterior and posterior segment inflammation within 48 to 72 h after tamponade placement. Common ocular findings included limbal corneal infiltrates, anterior chamber cells, vitritis, and decreased visual acuity. Three patients also experienced non-ocular symptoms, including perichondritis and auditory disturbances. Comprehensive evaluations, including infectious and autoimmune workups, were unremarkable in all cases. None of the patients had a prior history of uveitis or known systemic inflammatory conditions. Prompt initiation of topical and systemic corticosteroids led to the resolution of inflammation and recovery of visual function in all individuals. The uniform timing, bilateral occurrence of inflammation, and lack of alternative explanations indicate a possible association with the use of the Chitosan-based tamponade. The underlying mechanism remains uncertain, but an immunologic or toxic reaction is suspected.

**Conclusion:**

This case series highlights a possible association between intrauterine Chitosan tamponade and the onset of bilateral intermediate uveitis in postpartum patients. Although causality cannot be definitely established, the temporal clustering and consistent clinical presentation warrant caution and further investigation into the systemic immunogenic potential of Chitosan-based products.

## Background

Postpartum hemorrhage (PPH) remains one of the leading causes of maternal morbidity and mortality worldwide, accounting for approximately one-quarter of all maternal deaths [1]. Rapid and effective management of PPH is essential to prevent life-threatening complications [2]. In addition to pharmacological interventions such as oxytocin, misoprostol, and tranexamic acid a wide range of biophysical and surgical methods can be used to control intrauterine hemorrhage [3]. Among these, intrauterine tamponade techniques have become important tools for controlling hemorrhage when medical management alone is insufficient [4].

In recent years, hemostatic adjuncts such as Chitosan coated gauze (Celox^®^, MedTrade Products, UK) have been introduced into obstetric practice to aid in managing PPH. This has been particularly helpful when rapid intervention is necessary or conventional measures have failed [5]. The product mentioned here was originally developed for battlefield and trauma use since it effectively controls extensive arterial and venous hemorrhages [6]. Composed of chitosan—a natural polysaccharide derived from the shells of crustaceans—Celox^®^ forms a robust, adhesive gel-like clot upon contact with blood, acting independently of the body’s coagulation cascade. This unique property allows it to be used effectively even in individuals with coagulopathies or those receiving anticoagulant therapy [7].

In the context of obstetrics, Celox^®^ has been applied intrauterine as a gauze roll to control severe uterine bleeding during or after cesarean sections and vaginal deliveries. Several case reports and small observational studies have noted successful hemorrhage control using intrauterine Celox^®^ without the need for hysterectomy, suggesting a potential role in fertility-preserving interventions [5, 8, 9].

While its local hemostatic efficacy is well-documented the FDA and EMA product labeling for Celox does not list any ocular side effects [10, 11].

## Introduction

This case series presents six postpartum patients who developed acute bilateral intermediate uveitis shortly after intrauterine application of Celox^®^ gauze during cesarean section. Celox^®^ is a chitosan-based hemostatic dressing designed to induce coagulation through ionic interaction with erythrocyte membranes. All patients were previously healthy and exhibited rapid-onset photophobia, ocular pain, and visual disturbance within 48–72 h after tamponade placement. Ophthalmologic examination revealed diffuse, non-granulomatous intraocular inflammation involving both anterior and posterior segments. Infectious and autoimmune etiologies were excluded, suggesting a systemic inflammatory or toxic response related to Celox^®^ components. Although Chitosan is generally regarded as biocompatible, its intrauterine application in a highly vascular postpartum environment may facilitate systemic exposure and provoke an aberrant immunologic reaction. Thus far, only one comparable case report - interpreted as atypical Cogan’s syndrome [12] - has been published, highlighting the need for further study of the mechanisms and safety of intrauterine Chitosan tamponade.

We conducted a retrospective review of six postpartum patients admitted to a secondary care center between March and October 2023. All individuals developed bilateral ocular symptoms shortly after undergoing cesarean section with intrauterine Celox^®^ tamponade for PPH. Data collected included patient demographics, obstetric history, intraoperative findings, blood loss estimation, laboratory data (CRP, procalcitonin, coagulation parameters), systemic and topical treatments, and ophthalmological evaluations.

All patients underwent multiple ophthalmologic consultations and were followed clinically until resolution of symptoms. Infectious and autoimmune causes were evaluated including blood cultures, urinalysis, imaging and serological testing.

## Case presentations

### Case 1

A 42-year-old woman (gravida III, para II) underwent cesarean section at 38 + 4 weeks of gestation because of uterine atony, with an estimated intraoperative blood loss of 1200 mL. Hemostasis was achieved using an intrauterine Celox^®^ gauze tamponade. Forty-eight hours after placement, she developed bilateral photophobia, ocular pain, and marked visual deterioration. Ophthalmologic examination revealed 4 + anterior-chamber cells, a sterile hypopyon, dense vitritis (haze 4+), and circular limbal infiltrates; best-corrected visual acuity (BCVA) was reduced to hand motion in both eyes. The findings were consistent with acute bilateral intermediate uveitis with sterile hypopyon, presumed secondary to a systemic inflammatory reaction following intrauterine Celox^®^ exposure.

A comprehensive infectious and systemic work-up, including blood cultures, magnetic resonance imaging, urinalysis, and extensive serologic testing for bacterial, viral, and autoimmune causes, was negative. Laboratory evaluation showed markedly elevated inflammatory markers (C-reactive protein 395.3 mg/L, procalcitonin 65.6 ng/mL) and a mild Factor XIII deficiency. Systemic corticosteroid therapy was initiated with oral prednisolone.

1 mg/kg/day (60 mg total), together with hourly topical prednisolone acetate 2.5% and cyclopentolate 1% three times daily.

**Fig. 1:**
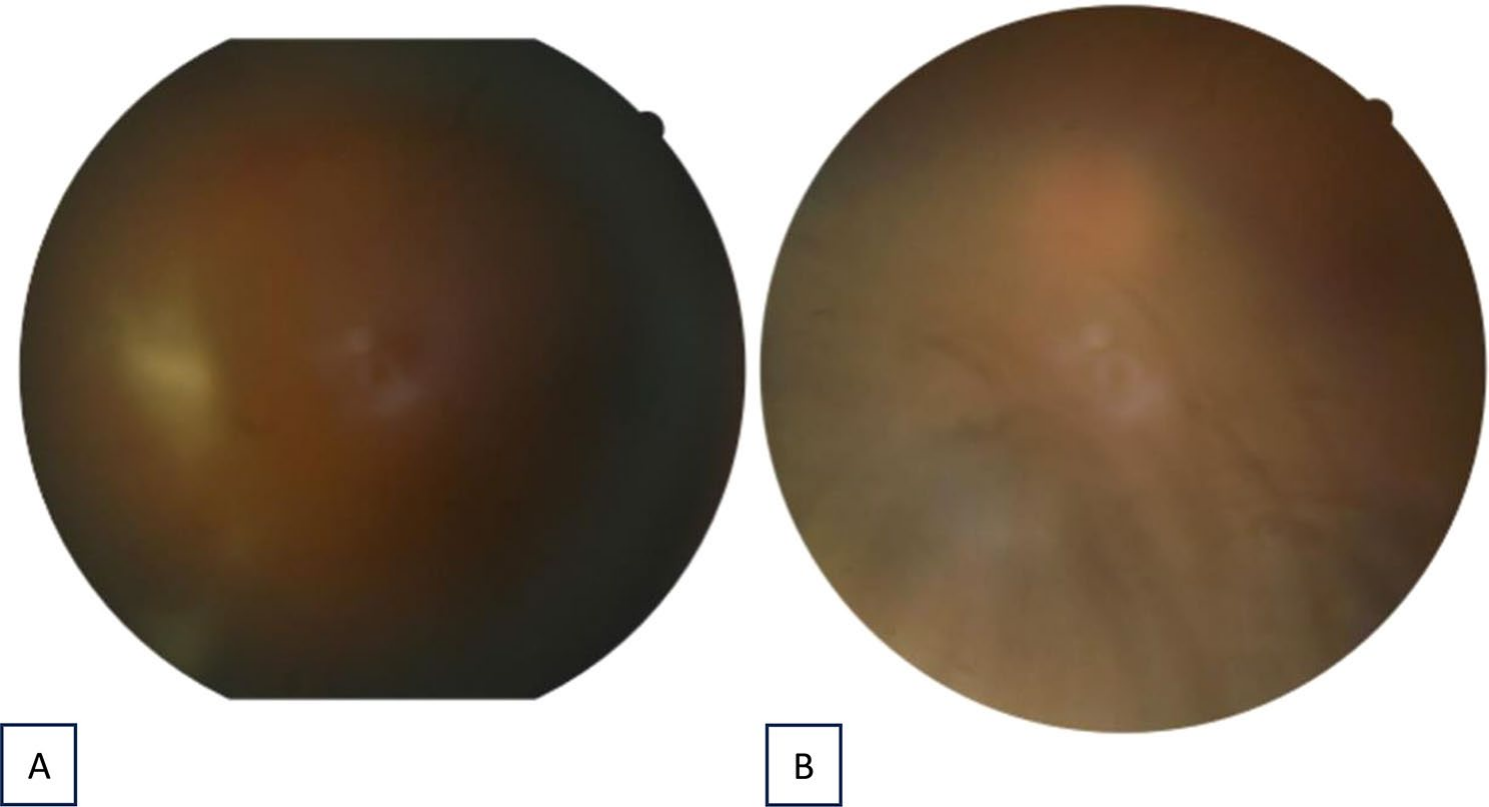
Fundus photography of the right eye (**A**) and the left eye (**B**) 3 days after initial treatment

**Fig. 2:**
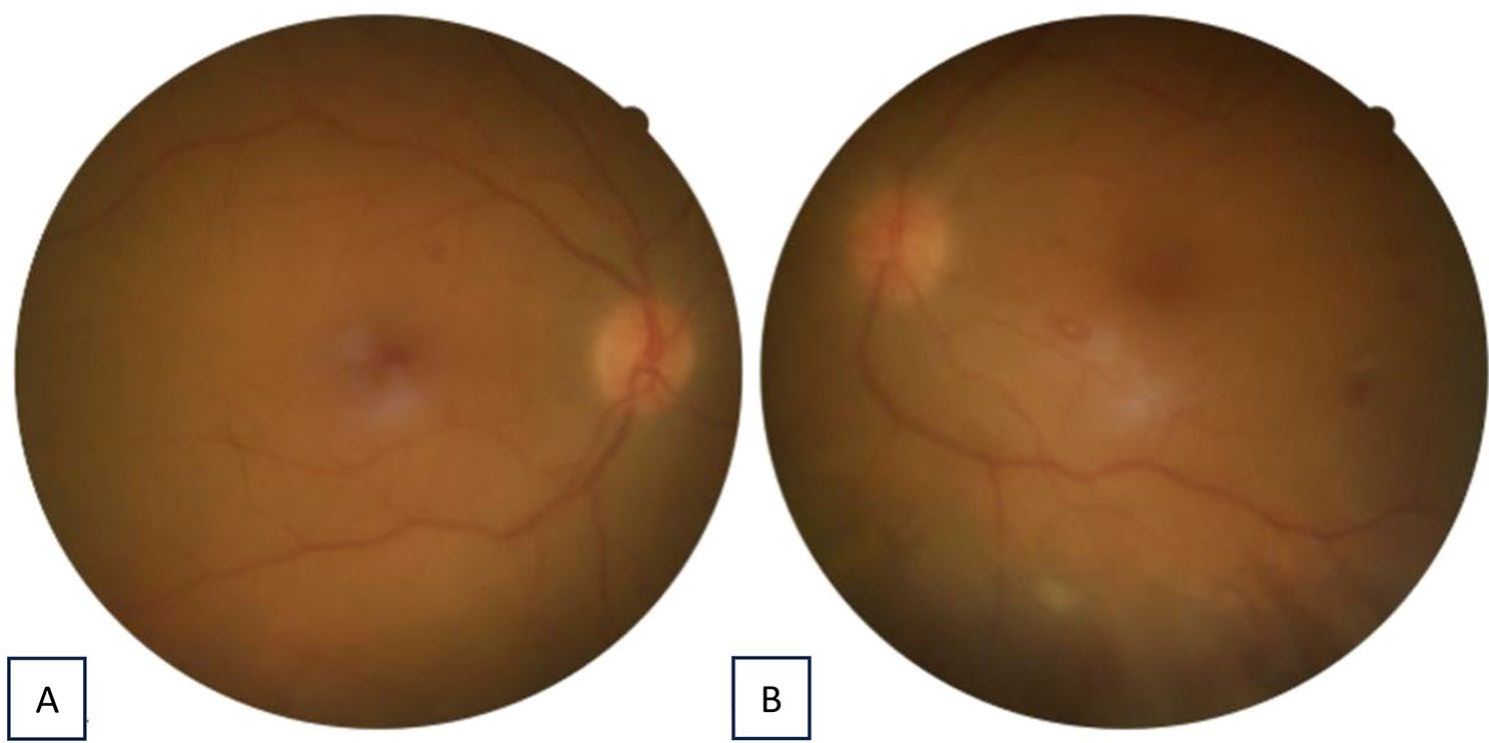
Fundus photography of the right eye (**A**) and the left eye (**B**) 5 days after initial treatment

Clinical improvement was first noted within three days of treatment initiation. Topical corticosteroids were tapered over two weeks and systemic therapy over four weeks. At the six-week follow-up, BCVA had improved to 0.6/0.8 (Snellen) bilaterally, with complete resolution of intraocular inflammation and no recurrence during subsequent visits (Figs. 1 and 2).

**Fig. 3:**
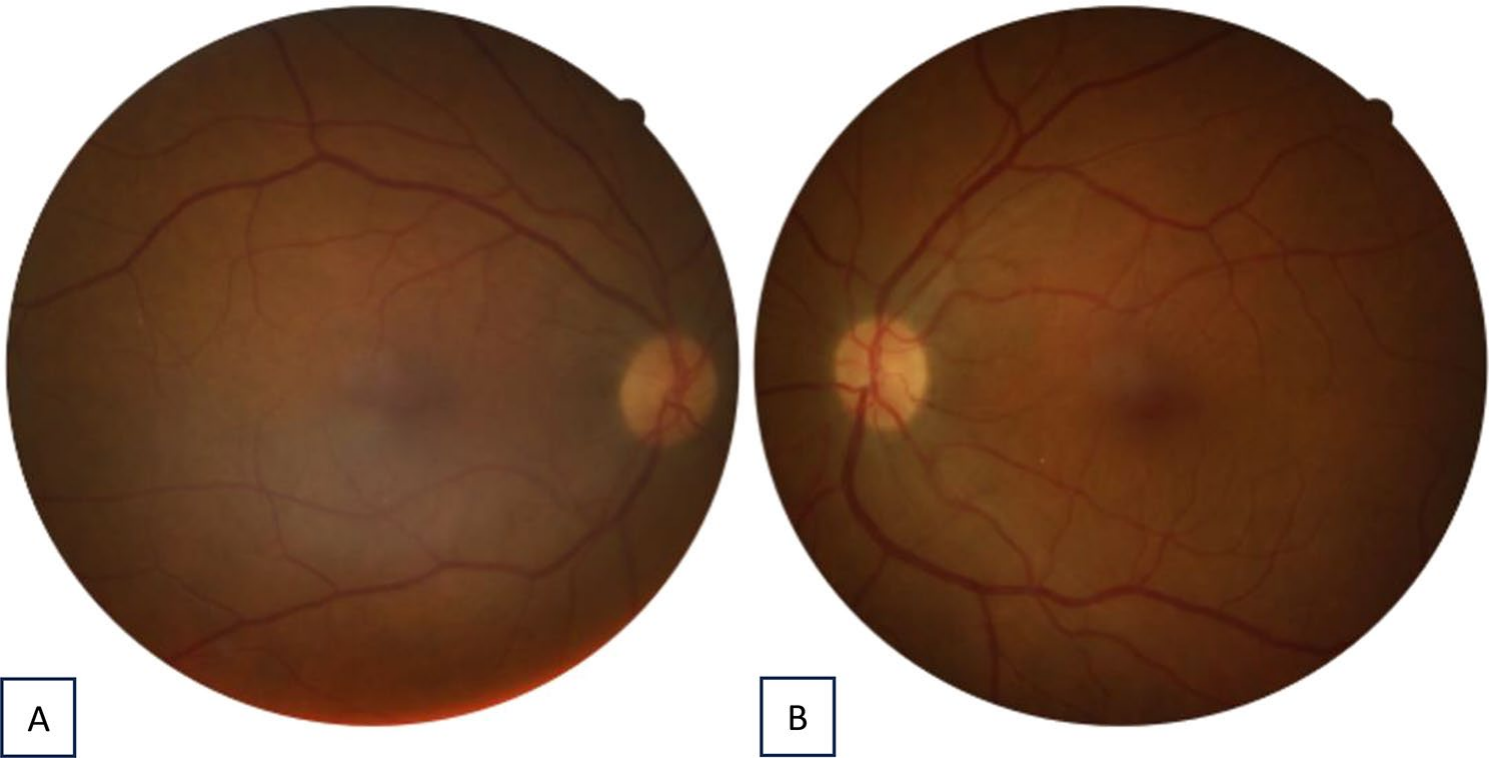
Fundus photography of the right eye (left) and the left eye (right) 3 weeks after initial treatment

**Fig. 4:**
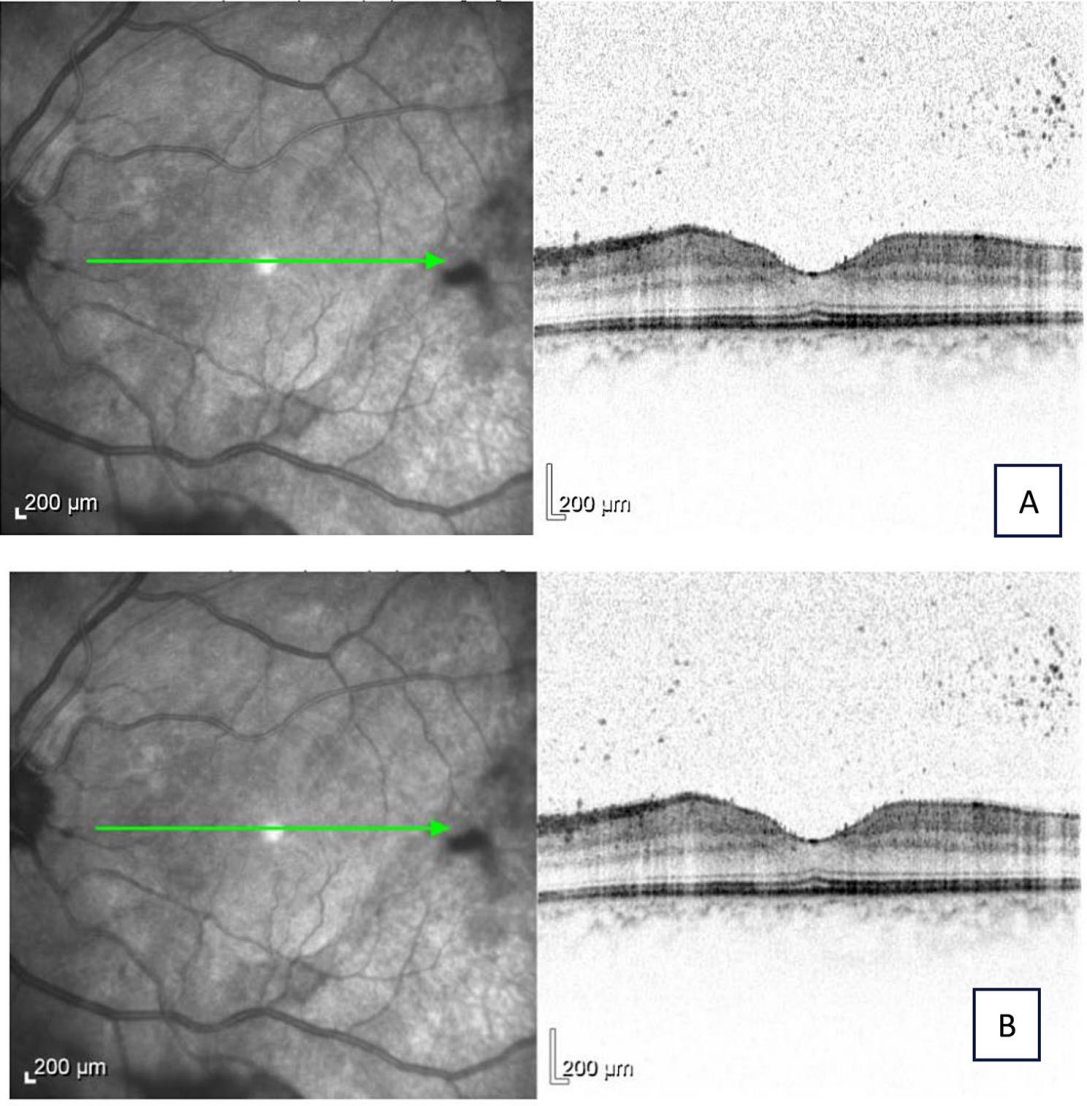
Macula OCT of the right eye (**A**) and the left eye (**B**) 3 weeks after initial treatment

### Case 2

A 23-year-old primigravida underwent cesarean section at 36 + 1 weeks due to fetal growth restriction and pathological cardiotocography. Following intraoperative blood loss of 1800 mL, a Celox^®^ tamponade was placed for hemostasis. Two days later, she developed bilateral blurred vision. Examination showed 3 + anterior chamber cells, limbal infiltrates, and vitritis in both eyes, with best-corrected visual acuity (BCVA) of 0.3 bilaterally. Infectious and autoimmune work-up was negative except for a mild reduction in Factor XIII activity, and C-reactive protein was elevated to 271.5 mg/L. A diagnosis of acute bilateral intermediate uveitis was made (Figs. 3 and 4).

Treatment with oral prednisolone 1 mg/kg/day (55 mg) and topical prednisolone acetate 2.5% hourly plus cyclopentolate 1% twice daily led to rapid improvement within three days and complete resolution by day 6, when BCVA had recovered to 1.0 in both eyes. Systemic steroids were tapered over three weeks, and no recurrence occurred during follow-up.

### Case 3

A 33-year-old woman (gravida VII, para IV) with a history of multiple cesarean sections underwent emergency surgery at 31 + 4 weeks of gestation for placenta previa and uterine rupture. Intraoperatively, Celox^®^ gauze was applied to control bleeding, followed by hysterectomy and bladder repair. Two days later, she developed bilateral ocular discomfort and blurred vision. Ophthalmologic examination revealed fine keratic precipitates, 2 + anterior chamber cells, and mild vitritis, with best-corrected visual acuity (BCVA) of 0.5 in both eyes. Concurrently, she exhibited perichondritis and auditory disturbances.

Extensive infectious and autoimmune testing was negative. Coagulation analysis showed elevated factors VIII and IX, interpreted as an acute-phase response. A diagnosis of acute bilateral intermediate uveitis was made. Treatment consisted of oral prednisolone 60 mg daily and topical prednisolone acetate 2.5% hourly. Clinical improvement occurred within three days, with complete resolution of inflammation after ten days. Corticosteroids were tapered over four weeks, and no relapse occurred during follow-up. The associated perichondritis and auditory symptoms subsided completely in parallel with ocular improvement.

### Case 4

A 39-year-old woman (gravida VI, para II) underwent cesarean section at 37 + 1 weeks for placenta previa and a thin lower uterine segment. Intraoperatively, Celox^®^ gauze tamponade was used to control bleeding after an estimated blood loss of 2300 mL. Two days postpartum, she developed bilateral blurred vision and ocular discomfort. Ophthalmologic examination showed bilateral anterior uveitis with 2 + anterior chamber cells and fine keratic precipitates; best-corrected visual acuity (BCVA) was 0.8 in the right eye and 1.0 in the left eye.

Laboratory testing revealed a markedly elevated C-reactive protein of 321.5 mg/L, declining rapidly with treatment. No infectious or autoimmune cause was identified. The patient received oral prednisolone 70 mg daily, topical prednisolone acetate 2.5% hourly, and cyclopentolate 1% twice daily. First improvement was noted after two days of therapy. Systemic corticosteroids were maintained for seven days at full dose and tapered over the following three weeks. At the four-week follow-up, BCVA was 1.0 in both eyes with complete resolution of inflammation and no recurrence.

### Case 5

A 39-year-old primigravida underwent cesarean section at 34 + 1 weeks of gestation for vaginal bleeding and uterine atony, with an estimated blood loss of 1500 mL. Intrauterine Celox^®^ gauze was applied to achieve hemostasis. Two days later, the patient reported bilateral eye pain and vision loss. Ophthalmologic examination revealed 3 + anterior chamber cells, vitritis, and limbal nodules in both eyes, with BCVA of 0.4 bilaterally. A small peripapillary retinal hemorrhage was also observed.

Laboratory testing showed elevated inflammatory markers (C-reactive protein 185.4 mg/L), while infectious and autoimmune investigations were unremarkable. A diagnosis of acute bilateral intermediate uveitis was established. Treatment with oral prednisolone 1 mg/kg/day (65 mg total), topical prednisolone acetate 2.5% hourly, and cyclopentolate 1% twice daily led to marked improvement within four days. Systemic corticosteroids were tapered over four weeks. At the one-month follow-up, BCVA had fully recovered to 1.0 in both eyes, the retinal hemorrhage had resolved, and no recurrence was observed.

### Case 6

A 31-year-old primigravida underwent cesarean section at 36 + 2 weeks of gestation for placenta previa totalis. Due to postpartum hemorrhage with an estimated blood loss of 1000 mL, a relaparotomy was performed, and intrauterine Celox^®^ gauze tamponade was placed. Two days later, she presented with bilateral ocular pain, photopsia, and blurred vision. Ophthalmologic examination showed bilateral intermediate uveitis with anterior chamber cells, vitreous haze, and dense vitreal opacities. Optical coherence tomography confirmed preserved foveal structure and epiretinal gliosis in the left eye. Concomitant perichondritis, hand joint swelling, and hypoacusis were also noted.

Systemic and infectious evaluations were negative. Oral prednisolone 1 mg/kg/day and topical prednisolone acetate 2.5% hourly were initiated, leading to marked improvement within five days. Topical therapy was tapered gradually as inflammation subsided, and systemic corticosteroids were discontinued after four weeks. At the six-week follow-up, best-corrected visual acuity was 1.0 in both eyes, and all extraocular symptoms, including perichondritis and auditory disturbances, had completely resolved (Table 1).

**Table 1:** Summary of relevant findings on the birth process, clinical ophthalmological assessments and selected laboratory parameters

Case	Age (years)	Gravida/ Para	Indication for C-section	Blood Loss (mL)	Ocular Findings	CRP (mg/L)	Treat ment	Outcome
1	42	G3P2	Preterm rupture of membranes	1200	Bilateral 4 + AC cells, Dense vitritis, hypopyon, limbal infiltrates	395.3	Oral prednisolone 60 mg/day, topical prednisolone acetate 2.5% hourly, cyclopentolate 1% TID	VA improved to 0.6/0.8 bilaterally; no recurrence
2	23	G1P0	Preterm rupture of membranes, pathological cardiotocography (CTG), fetal growth restriction	1800	Bilateral uveitis, limbal infiltrates, vitritis	271.5	Oral prednisolone 55 mg/day, topical prednisolone acetate 2.5%, cyclopentolate 1% BID	VA restored to 1.0 bilaterally in 6 days
3	33	G7P4	Premature contractions and placenta accreta spectrum	Not specified	Bilateral anterior uveitis, mild vitritis, endothelial precipitates		Oral prednisolone 60 mg/day, topical prednisolone acetate 2.5% hourly	Resolution in 10 days, no relapse
4	39	G6P2	Placenta previa totalis and impending uterine rupture	2300	Bilateral anterior uveitis, 2 + cells, keratic precipitates	321.5	Oral prednisolone 70 mg/day, topical prednisolone acetate 2.5%, cyclopentolate 1% BID	VA 1.0/1.0, CRP normalized, no recurrence
5	39	G1P0	Preterm contractions, preterm premature rupture of membranes with placental abruption, pathological CTG	1500	Bilateral uveitis, 3 + AC cells, vitritis, limbal nodules, retinal hemorrhage	185.4	Oral prednisolone 65 mg/day, topical prednisolone acetate 2.5%, cyclopentolate 1% BID	VA improved to 1.0/1.0, CRP declined, no relapse
6	31	G1P1	Placenta previa totalis	1000	Bilateral uveitis, AC cells, vitritis	223,3	Oral prednisolone 65 mg/day, topical prednisolone acetate 2.5%, cyclopentolate 1% BID	VA improved to 1.0/1.0, CRP declined, no relapse

## Discussion

This case series reports a unique observation of acute bilateral uveitis following the use of intrauterine Chitosan coated gauze (Celox^®^) tamponade. While causality cannot be definitively established, the consistent temporal relationship across six otherwise unrelated cases strengthens the suspicion of an immunologic or toxicologic mechanism.

Celox^®^ is composed primarily of oxidized regenerated cellulose, designed to act as a mechanical and biochemical hemostat by promoting coagulation [13]. In surgical fields, it is generally absorbed locally with minimal systemic absorption. However, intrauterine placement — especially in the highly vascular postpartum uterus — may allow systemic dissemination of breakdown products. Hypothetically, these could trigger a systemic inflammatory or immune-mediated response.

The simultaneous bilateral ocular involvement, absence of infectious etiology, and favorable response to corticosteroids point toward a non-infectious inflammatory process. The presence of corneal limbal infiltrates and posterior segment involvement is rare in classic uveitis and may underline the suspected hypersensitivity or toxic mechanism.

All six patients were managed with a combination of topical and systemic corticosteroids, initiated promptly upon diagnosis of intermediate or anterior uveitis. The standard regimen included:

- Oral prednisolone at 1 mg/kg/day, initiated within 24 h of diagnosis. Duration of high-dose systemic steroids ranged from 5 to 7 days, followed by tapering over 3–4 weeks depending on clinical response and inflammatory markers.
- Topical corticosteroids, primarily prednisolone acetate 2.5%, applied hourly during acute inflammation, then tapered to 2–4 times daily over 1–2 weeks.
- Cycloplegic agents such as cyclopentolate 1% were used to prevent posterior synechiae and reduce ciliary spasm. These were typically prescribed BID or TID for 1–2 weeks.

This aggressive yet standardized approach was effective in reversing inflammation in all patients without the need for intravitreal corticosteroids or immunomodulatory therapy with other agents. Importantly, no patients experienced steroid-induced ocular hypertension or systemic complications, and all had complete or near-complete recovery of visual acuity.

The consistent favorable response to corticosteroids supports a non-infectious inflammatory etiology as well [14]. It further strengthens the hypothesis of Celox^®^-associated systemic immune activation or toxicity rather than an infectious process. All six patients had no evidence of systemic autoimmune disease on serologic testing.

Differential diagnoses for postpartum uveitis include infectious endophthalmitis, autoimmune uveitis, and paraneoplastic syndromes [15]. However, none of the patients had prior autoimmune conditions or infectious sources to explain the bilateral involvement. Extensive testing, including blood cultures, serologies, and imaging, consistently failed to identify an alternate cause.

HELLP syndrome and preeclampsia-related retinopathy were also ruled out due to the absence of systemic features necessary for the diagnosis of such diseases. Drug-induced uveitis from medications were considered, but no specific pattern emerged across cases except for Celox^®^.

A recently published case series including three individuals reported similar ocular findings such as bilateral intermediate uveitis with systemic involvement following cesarean sections and the use of Celox^®^ temponade [12]. Affected patients developed severe intraocular inflammation, with some experiencing hearing loss and otologic symptoms as well. The close temporal association, and improvement after Celox^®^ removal in one case, suggested a possible immunogenic trigger. The authors interpreted the constellation of clinical symptoms as an atypical Cogan’s syndrome, a rare chronic inflammatory condition with ocular and vestibuloauditory symptoms. Similarly, our patients developed intraocular inflammation, some auditory symptoms and perichondritis shortly after Celox^®^ exposure. However, none progressed to further systemic involvement, which may be attributable to early initiation of systemic corticosteroid therapy. These observations support a potential association between chitosan-based hemostatic agents and immune-mediated ocular or even systemic inflammation. It may be speculated that the involvement of immune-privileged sites—such as the eye, the central nervous system, and the pregnancy-dependent immune privilege of the uterus—could connect these events.

Some rare case reports have associated other hemostatic agents or surgical materials with systemic inflammatory syndromes, which are involved in the process of releasing inflammatory cytokines, but ocular inflammation has not been described [16]. The FDA and EMA product labeling for Celox does not list ocular side effects at all.

This cluster may represent an unrecognized complication, possibly underreported due to often mild symptoms or lack of systematic ophthalmologic evaluation in postpartum patients.

This case series has several limitations. Its retrospective nature and small sample size preclude definitive causality. Other unmeasured variables such as host immune status, co-medications, or subtle surgical complications could have contributed. No biopsy or histopathology was performed. Nonetheless, the reproducibility of findings across patients with different surgeons and circumstances is noteworthy.

## Conclusion

We present a case series of six postpartum patients who developed mild up to severe bilateral intermediate or anterior uveitis following intrauterine Celox^®^ tamponade use after cesarean section. The rapid onset, bilateral nature, lack of infections, and favorable corticosteroid response strongly suggest a systemic inflammatory or immune-mediated mechanism potentially related to Celox^®^ exposure. Obstetricians should be aware of this possible complication and consider early ophthalmologic or ENT evaluation in patients presenting with visual or auditory symptoms postoperatively.

Further investigations, including pharmacovigilance studies and experimental models, are warranted to determine the safety profile of intrauterine Celox^®^ in the postpartum setting.

## Data Availability

The datasets generated and/or analyzed during the current study are not publicly available due to patient confidentiality but are available from the corresponding author on reasonable request.

## Abbreviations

PPH: Postpartum Hemorrhage
FDA: U.S. Food and Drug Administration
EMA: European Medicines Agency
CRP: C-Reactive Protein
AC: Anterior Chamber
MRI: Magnetic Resonance Imaging
OCT: Optical Coherence Tomography
VA: Visual Acuity
CTG: Cardiotocography
BID: Bis in die (twice daily)
TID: Ter in die (three times daily)
HELLP: Hemolysis, Elevated Liver enzymes, Low Platelets
